# 疫情防控常态下肺癌早筛的现状及进展

**DOI:** 10.3779/j.issn.1009-3419.2020.101.47

**Published:** 2021-01-20

**Authors:** 郁杨 王, 娜 周, 栋 刘, 晓春 张

**Affiliations:** 1 266000 青岛，青岛大学医学部 Department of Medicine, Qingdao University, Qingdao 266000, China; 2 266000 青岛，青岛大学附属医院 The Affiliated Hospital of Qingdao University, Qingdao 266000, China

**Keywords:** 疫情防控, 肺肿瘤, 早期筛查, Epidemic prevention and control, Lung neoplasms, Early screening

## Abstract

肺癌是我国发病率最高的恶性肿瘤。早发现早鉴别出有症状的肺癌患者和及时从高危人群中筛选出无症状患者需要多方面配合。目前，尽管已经联合影像学、血清学、基因组学、蛋白质组学等手段对可疑肺癌进行筛查，但仍存在漏诊、误诊等问题。同时，新型冠状病毒肺炎（corona virus disease 2019, COVID-19）流行病的蔓延给肺癌早筛带来了新的挑战。在疫情防控常态化下，肺癌早筛工作应该做出相应改变：提高人群防癌控癌意识、加强就诊流程管理、提高肿瘤检出效率、优化检测技术并合理利用互联网和大数据平台。联合多种筛查方法建立一种理想的肺癌早筛模式，保证疫情防控常态下肺癌早筛工作既精简又高效。

## Abstract (en)

Lung cancer is the malignant tumor with the highest incidence in China. Early detection and identification of symptomatic lung cancer patients and timely screen out asymptomatic patients from high-risk groups require multiple cooperation. At present, although combined imaging, serology, genomics, proteomics and other methods have been combined to screen for suspected lung cancer, there are still problems such as missed diagnosis and misdiagnosis. Meanwhile, the spread of the corona virus disease 2019 (COVID-19) epidemic has brought new challenges to early lung cancer screening. Under the normalization of epidemic prevention and control, the work of early lung cancer screening should be changed accordingly: improve the population's awareness of cancer prevention and control, strengthen the management of medical procedures, improve the efficiency of tumor detection, optimize detection technology, and utilize internet and big data platforms rationally. We should establish an ideal model, combining multiple screening methods, which is streamlined and efficient for early lung cancer screening under normal epidemic prevention and control.

新型冠状病毒肺炎（corona virus disease 2019, COVID-19）流行病的蔓延，极大地影响了常规医疗，也对肺癌早筛的进程产生了一定程度的影响。部分具有可疑肺癌早期症状的患者，为避免疫情防控下繁琐的筛查流程，仅购买非处方药物控制症状，延误了肺癌的早期诊疗。鉴于此，需要更便捷、高效的肺癌早筛技术的支持。在这篇综述中，我们重点讨论了新型冠状病毒肺炎疫情防控常态下肺癌高危人群筛查策略及技术的现状和进展。

## 1 新型冠状病毒肺炎疫情防控下有症状患者早筛策略

肺癌与疑似新型冠状病毒肺炎患者的临床表现有诸多相似之处，在排除新型冠状病毒肺炎诊断的同时，不能忽视其罹患肺癌的可能，高危人群的院内筛查是必要的。

### 1.1 新型冠状病毒肺炎患者临床表现

不同地区新型冠状病毒肺炎的症状大同小异，发热、咳嗽咳痰、胸闷和呼吸困难是常见表现。国内的一项回顾性研究中^[1]^，141例COVID-19患者中发热（> 37.5 ℃）139例（98.58%）、咳嗽106例（75.18%）、头痛11例（7.80%）、咳痰41例（29.08%）、胸闷93例（65.96%）、呼吸道以外症状主要有腹泻等4例（2.84%）。武汉金银滩医院99例患者中^[2]^，发热82例（82.83%），咳嗽81例（81.82%），呼吸困难31例（31.31%）。分析中国大陆30个省、自治区、直辖市的552家医院中1, 099例新型冠状病毒肺炎患者的数据^[3]^也提示发热与咳嗽是最常见症状，其中入院时发热占比43.8%，住院时发热占比88.7%，咳嗽占比67.8%。

### 1.2 肺癌患者临床表现

中央型肺癌的最常见症状是咳嗽、咯血、胸闷，肿瘤压迫位置不同也会造成声音嘶哑和呼吸困难。周围型肺癌根据肿瘤大小、位置、病理类型、分期等的不同也会表现出不同症状，包括肺内、肺外转移以及全身症状。一项问卷调查^[4]^分析了在6个月或12个月内经历单个或多个呼吸警报症状与肺癌诊断之间的关系，最终发现与肺癌明确相关的症状包括持续4周以上的咳嗽、食欲减退、呼吸困难或声音嘶哑。然而，另一项研究^[5]^显示咳嗽、咳痰、呼吸困难和一般症状（食欲减退、乏力、体重减轻）对肺癌的预测值很低（0.4%-1.1%）。新型冠状病毒肺炎和肺癌的临床表现均无特异性，且两者呼吸道症状有一定程度相似，在疫情防控常态化下，尽早就医鉴别极为必要。

### 1.3 疫情防控下筛查管理

#### 1.3.1 严格的分级诊疗

症状轻微、疑有新型冠状病毒肺炎或新发肿瘤的患者，应先到当地医院进行相关检查（新型冠状病毒核酸检测、影像学、细胞学、血液学等）明确诊断，诊断明确且有条件治疗者可在初诊医院进行规范治疗；限于技术条件无法明确诊断或是病情严重者可以由初诊医院请上级医院线上会诊，或提前预约至上级医院就诊。携带必要的初诊医院检验检查结果、诊疗介绍等按约定就诊，避免人力、物力、财力的浪费。

#### 1.3.2 有序的门诊管理

门诊管理人员需按预约时间、就诊时间引导患者至规定区域等待就诊；严格限制就诊患者间的安全防护距离；力争“一患一陪”并提醒患者及家属全程佩戴口罩；增加导医人数和指示标志，引导患者至正确的诊室就诊，减少不必要院内走动；杜绝“加塞”现象，降低院内等待时间。

## 2 新型冠状病毒肺炎疫情防控下无症状人群早筛策略

以非小细胞肺癌（non-small cell lung cancer, NSCLC）为例，参考肿瘤原发灶-淋巴结-转移（tumor-node-metastasis, TNM）分期，将早期肺癌定义为可经手术根治的原位癌及Ia期-IIb期（Tis-T3N0M0）的患者。早期肺癌中，很大一部分人群并无临床表现，仅发生影像学改变或脱落细胞学中查到癌细胞。因此，对无症状肺癌高危人群的早筛工作尤为重要。美国国立综合癌症网络（National Comprehensive Cancer Network, NCCN）指南将肺癌高危人群定义为：年龄 > 40岁，至少合并以下一项危险因素者：①吸烟史；②被动吸烟、环境油烟吸入史；③职业暴露史（石棉、铍、铀、氡等接触者）；④有恶性肿瘤病史或肺癌家族史；⑤有慢性阻塞性肺疾病或弥漫性肺纤维化病史。中华医学会提出中国肺癌高危人群筛查标准包括：年龄在45岁-70岁、有吸烟史、存在有毒有害物质接触史（相关职业史）、恶性肿瘤家族遗传史的群体。应充分认识到年老体弱者在无症状高危人群中占比极大，疫情防控期间对此类人群的筛查工作应做出相应调整。

### 2.1 延迟活检时间

因疫情管控，所有送检组织与细胞标本都按照潜在感染样本处理，应尽量避免新鲜标本进入病理科。疑似肺癌病例应综合各检查结果评估其患癌风险，慎重决定是否可以延迟活检时间。并对延迟活检者密切随访，一旦发现进展及时处理。

### 2.2 延迟随诊时间

对于低风险肺小结节患者，减少不必要的有创检查，延长复查周期。对于已规律随访一段时间且病灶稳定者，疫情防控期间可以暂停复查。待疫情结束，对其继续随访。若降低随访频率和延长复查周期不会造成低风险小结节患者罹患肺癌的漏诊、误诊，可以考虑将此策略推广，以减少医疗资源浪费、减轻就医负担及减缓患癌焦虑。

### 2.3 社区筛选高危人群

疫情防控常态下，社区的第一步普筛工作对控制人员流动（特别是院内）尤为重要。以问卷调查的形式，在社区内筛选肺癌高危人群，确定需医院就诊的人员并建立随访表将会大大提高肺癌早筛的效率。例如，云南省开展的一项癌症早诊早治工程^[6]^，利用肺癌高危人群评估模型对昆明市4个主城区36个街道办事处165, 337人进行问卷调查及肺癌风险评估，评估为高风险者进行低剂量计算机断层扫描（low-dose computed tomography, LDCT）筛查，结果显示此模型的敏感度为43.94%（116/264），特异度为77.10%（127, 275/165, 073），筛查组早诊率为72.97%（54/74），明显高于非筛查组的28.48%（43/151）。同时，街道办事处及社区工作人员有必要进行定期的健康宣讲，以提高居民防癌控癌意识。

## 3 肺癌早筛技术

### 3.1 影像学检查

中华医学会肺癌临床诊疗指南（2020）以Ⅰ类证据推荐高危人群进行低剂量CT（low-dose CT, LDCT）筛查，LDCT以常规CT六分之一的放射剂量即能达到常规CT对肺癌筛查的敏感性。早在2010年，美国肺癌筛查试验（The National Lung Screening Trial, NLST）数据^[7]^显示，在一组肺癌高危成年人中，随机接受3次连续年度LDCT肺癌癌症筛查检查（基线、1年和2年）的肺癌死亡率降低了20%。一项国内研究^[8]^分析比较了LDCT和常规CT图像质量和辐射剂量，两组在图像质量及肺癌和肺小结节检出率上差异明显（*P* < 0.05），在辐射剂量上无明显差异（*P* > 0.05）。作为肺癌早筛最重要手段，LDCT也存在缺陷：过度诊断、假阳性率高、费用高昂等。研究^[9]^显示LDCT检出肺癌的灵敏度为93.8%，但特异度仅为73.4%，性质不明的肺小结节、肺结核或肺内非特异性炎症可能被误判。对于LDCT可疑肺癌的患者，结合高分辨率CT、正电子发射计算机断层显像（positron emission tomography, PET）将提升早筛精准度。

### 3.2 血液检测

#### 3.2.1 肿瘤相关抗原

临床常用于肺癌诊断与疗效评价的血浆蛋白标志物包括癌胚抗原153、鳞状细胞癌抗原、细胞角蛋白片段21-1、特异性神经元烯醇酶和促胃泌素原释放肽。一项研究^[10]^对肺癌高风险人群进行肿瘤抗原联合检测，诊断肺癌的特异度高达96%，但灵敏度仅为49%。需要注意，肿瘤相关抗原血浆稳定性差且在早期肺癌中阳性率低^[11]^，不能作为单一的筛查手段，也不能忽略肿瘤相关抗原阴性者。

#### 3.2.2 肺癌自身抗体

肺癌自身抗体针对肺癌抗原产生，其特异度高且出现于影像学检出病灶前，适于肺癌早筛。一项临床研究^[12]^评估了联合检测肺癌自身抗体p53、NY-ESO-1、CAGE、GBU4-5、SOX2、HuD和MAGE A4对1, 613例肺癌患者的诊断效能，灵敏度为41%，特异度为87%。另一项针对中国人群的研究^[13]^提出LDCT联合自身抗体对早期肺癌的检出率可达95%。肺癌自身抗体稳定性好、半衰期长，检测技术简便易行，是对肿瘤相关抗原检测很好的补充手段。

#### 3.2.3 循环肿瘤细胞（circulating tumor cells, CTCs）

CTCs是从肿瘤脱落并进入循环系统的肿瘤细胞。最近的一项前瞻性、多中心研究^[14]^验证了叶酸受体（folate receptor, FR）在肺癌组织中高表达，肺癌患者中FR^+^的CTCs（FR^+^ CTCs）水平显著高于良性肺疾病者，FR^+^ CTCs用来鉴别良恶性肺结节的灵敏度为78.6%-82.7%、特异度为68.8%-78.4%。这都为CTCs在肺癌早筛的应用提供了理论支持。但外周血中的CTCs占比低，对检测技术的灵敏度要求极高。

#### 3.2.4 循环肿瘤细胞DNA

循环肿瘤细胞DNA（circulating tumor DNA, ctDNA）是一种广谱肿瘤标志物，是凋亡或死亡肿瘤细胞DNA、活动期肿瘤细胞、CTCs的总和^[15]^，在肿瘤早期即出现。最近的一项研究^[16]^纳入192例肺部占位性病变（136例恶性和56例良性），血浆ctDNA的总体敏感度为69%，特异度为96%，对比血清蛋白检测（敏感度为51%，特异度为83%）有显著提高；联合ctDNA突变和血浆生物标志物将灵敏度和特异度分别提高到80%和99%。ctDNA检测未能广泛应用的主要原因有以下几点：①突变类型无法明确指示肿瘤类型；②肺癌异质性大、突变谱广，测序基因难以选择；③ctDNA的血浆浓度与肿瘤负荷相关，Ⅰ期肺癌中灵敏度只有50%，测序深度需加强^[17]^；④检测成本高。

#### 3.2.5 DNA甲基化水平

一项研究^[18]^表明矮小同源盒基因（short stature homeobox protein 2, *SHOX2*）超甲基化有望成为诊断肺癌的标志物之一，其鉴别肺癌与肺良性疾病的灵敏度为68%，特异度为95%。此研究标本取材于肺癌组织，早期肺癌痰液标本及血液中*SHOX2*超甲基化水平低于肿瘤组织，这对敏感性提出了极高的要求。此研究也提示我们关注基因甲基化水平在肺癌诊断中的价值。

#### 3.2.6 微小RNA分子（microRNA, miRNA）

miRNA广泛参与肺癌进程，一项研究^[19]^认为miRNA-146b、miRNA-205、miRNA-29c和miRNA-30b对NSCLC具有出色的诊断能力[受试者工作特征曲线下面积（area under the curve, AUC）为0.99，准确度为95.00%]，且相对于鳞状细胞癌（AUC为0.93，灵敏度为90.32%）对腺癌（AUC为0.98，灵敏度为99.10%）具有相对更高的预测能力和敏感性。之前的一项研究^[20]^还发现miRNA和肿瘤组织类型相关，可以通过血清中has-miRNA-025的表达水平区分肺腺癌与肺鳞癌，准确率达100%，可以弥补ctDNA对肿瘤类型诊断不清的劣势^[21]^。然而，需要注意的是，miRNA也在肺癌相关炎性疾病、肺纤维化或其他癌症中发生变化。目前，检测miRNA仍缺少标准化的分析参数、临床生物学信息和所有合并症的数据库，因此无法保证诊断早期肺癌的特异性。

### 3.3 遗传易感基因

一项研究^[22]^对29, 266例正常人和56, 450例肺癌患者进行全基因组关联研究（genome-wide association studies, GWAS）分析，筛选出10个肺癌遗传易感基因。其中，4个基因与肺癌（所有类型）相关，6个基因与肺腺癌相关，突显了肺癌组织学亚型在遗传易感性上的显著异质性。对普通人群进行遗传易感基因筛查，可以在早诊的同时兼顾早防，实现更加精准的个体化早防早诊早治。当然，这些新的肺癌易感基因在肺癌筛查中的价值尚待证实，其昂贵的检查费用也是目前无法普及的主要原因。

### 3.4 痰液筛查

痰脱落细胞学检查是肺癌早筛的重要手段，从细胞形态学识别上发现早期肺癌，联合荧光原位杂交技术（fluorescence *in situ* hybridization, FISH）提高检出率。一项研究^[23]^通过FISH检测痰标本中的染色体变化（缺失或扩增），其灵敏度为85.5%[95%置信区间（confidence interval, CI）：76.1%-92.3%]，特异度为69%（95%CI: 49.2%-84.7%），准确度为81.3%（95%CI: 72.8%-88.0%），阳性和阴性预测值分别为88.8%和62.5%。虽然FISH提高了肺癌早筛的敏感性，但提升程度有限，期待未来有新的前瞻性研究来评估FISH检测用于肺癌早筛的临床价值。

### 3.5 支气管镜筛查

对于可疑中央型肺癌、反复血痰但其他检测阴性的患者可以酌情选择支气管镜辅助筛查。目前的自荧光支气管镜（autofluorescence bronchoscopy, AFB）联合荧光共聚焦显微镜（fibered confocal fluorescence microscopy, FCFM）能更准确地观察到支气管壁形态结构，发现原位癌或癌前病变，必要时进行直视下活检。

### 3.6 大数据平台及风险评估模型

#### 3.6.1 蛋白组癌症早筛平台

肿瘤细胞在无限增殖的过程中可以表达出几百上千万拷贝的相关肿瘤蛋白，对肺癌样本中肿瘤蛋白进行检测及定量分析，建立蛋白组库，与健康人群的蛋白组相比较，筛选出有差别的蛋白质以指导肺癌的诊断。最近一项研究^[24]^借助Q-Exactive质谱仪，比较健康人和卵巢癌患者的子宫液囊泡中约3, 000种蛋白，筛选并验证卵巢癌特异蛋白质组（灵敏度为70%，特异度为76.2%）。相较肿瘤基因组，肿瘤蛋白质组出现时间更早、拷贝数量更多，且灵敏度明显优于基因检测。但目前针对肺癌的蛋白质组平台仍显单薄，尚需完善。

#### 3.6.2 恶性风险评估模型

针对中国肺癌高风险人群进行的一项大样本、多中心研究^[25]^，将肿瘤相关抗原与临床信息相结合构建肺癌风险模型，对早期肺癌有良好的预测价值（AUC为0.915）。另有李运模型^[26]^将肺结节患者临床特征及影像学特征作为独立影响因素纳入，对肺结节有很好的阳性预测率。恶性风险评估模型经济、便捷、客观，但其在独立因素的质量控制上良莠不齐。另外，仅将临床特征、影像学特征作为独立因素制作模型也限制了诊断的精确性和普适性。

## 4 小结与展望

肺癌早筛以LDCT筛查为主要手段，高度可疑阳性的患者采用有创病理穿刺与无创PET、肺癌抗原及自身抗体检测、细胞生物学检查等提高灵敏度和特异度。目前新型冠状病毒肺炎疫情防控常态化下，肺癌早筛应兼顾筛查技术的创新和筛查策略的调整。新型冠状病毒肺炎与早期肺癌患者的临床表现需要尽快鉴别，保证有症状人群及时就医；同时，对于无症状肺癌高危人群的筛查工作也不能松懈，合理的社区健康宣讲及初筛、优化的院内就诊流程、综合全面且互补的诊断方法应相互配合。新兴的筛查手段如ctDNA、CTCs、miRNA、DNA甲基化水平、数据信息平台等应进一步优化检测技术、规范技术标准、降低检测成本。结合传统的基础检测技术与新兴的精准检测技术，创建理想的肺癌早筛模式以期提高检出率和早诊率。在未来，通过互联网对被筛查者的临床资料、检测结果进行跟踪式管理，增加医患互动，制定出更加精准、更加个体化的肺癌早防早诊早治方案。
